# Visual short-term memory capacity predicts the “bandwidth” of visual long-term memory encoding

**DOI:** 10.3758/s13421-019-00954-0

**Published:** 2019-06-24

**Authors:** Keisuke Fukuda, Edward K. Vogel

**Affiliations:** 1 Department of Psychology, University of Toronto Mississauga, 3359 Mississauga Rd., Mississauga, ON L5L 1C6 Canada; 2 Department of Psychology, University of Chicago, Chicago, IL USA

**Keywords:** Memory models, Individual differences, Visual long-term memory, Visual short-term memory, Visual working memory

## Abstract

We are capable of storing a virtually infinite amount of visual information in visual long-term memory (VLTM) storage. At the same time, the amount of visual information we can encode and maintain in visual short-term memory (VSTM) at a given time is severely limited. How do these two memory systems interact to accumulate vast amount of VLTM? In this series of experiments, we exploited interindividual and intraindividual differences VSTM capacity to examine the direct involvement of VSTM in determining the encoding rate (or “bandwidth”) of VLTM. Here, we found that the amount of visual information encoded into VSTM at a given moment (i.e., VSTM capacity), but neither the maintenance duration nor the test process, predicts the effective encoding “bandwidth” of VLTM.

Although visual long-term memory (VLTM) has large enough capacity to store a virtually infinite amount of visual information (Brady, Konkle, Alvarez, & Oliva, 2008; Standing, 1973), not every information that we wish to remember is encoded. What limits our access to the unlimited memory storage? According to Atkinson and Shiffrin’s influential modal model of memory (Atkinson & Shiffrin, 1968, 1971; Rundus & Atkinson, 1970; Shiffrin & Atkinson, 1969), information is first encoded into short-term memory (STM) in which information is actively maintained. And this active maintenance is what grants access to long-term memory (LTM) storage. Despite its elegant simplicity, this model later received criticisms particularly on the proposed role of the STM maintenance in LTM encoding (Craik & Watkins, 1973; Naveh-Benjamin & Jonides, 1984). This criticism led to a discovery of the importance of the nature of encoding processes that information undergoes (Craik, 1983; Craik & Lockhart, 1972; Craik & Tulving, 1975; Craik & Watkins, 1973; Fisher & Craik, 1977; Moscovitch & Craik, 1976) and the characterization of the interaction between the encoding and retrieval processes became a central theme of LTM research. As a result, one aspect of LTM encoding proposed in the modal model—namely, its capacity limitation—received little attention to this date. That is, is there a capacity limitation in the amount of information encoded into LTM at a given time? If so, is this initial encoding bottleneck analogous to STM capacity? Recent studies that examined the limit of visual memory encoding had participants remember one object at a time, and therefore, their results do not directly inform us about the existence of such encoding bottleneck (e.g., Brady et al., 2008; Endress & Potter, 2014). In order to fully characterize the mechanism of VLTM encoding, it is important to examine whether there exists a capacity-limited encoding bottleneck by directly manipulating the amount of information that needs to be encoded into VLTM at the same time. Here, by manipulating the number and the quality of visual information that needs to be encoded into VLTM simultaneously, we found that VSTM capacity predicts the “bandwidth” of VLTM encoding due to a shared encoding bottleneck.

## Individual difference approach to examine the influence of VSTM capacity on encoding of VLTM

VSTM allows us to actively represent a limited amount of visual information in mind at a given time (Cowan, 2001; K. Fukuda, Awh, & Vogel, 2010a; Luck & Vogel, 2013). Although it is currently under debate as to how we should best characterize this capacity limitation (K. C. S. Adam, Vogel, & Awh, 2017; Bays & Husain, 2008; Fougnie, Asplund, & Marois, 2010; Luck & Vogel, 1997; Ma, Husain, & Bays, 2014; Rouder et al., 2008; van den Berg & Ma, 2018; Wilken & Ma, 2004; Zhang & Luck, 2008), researchers agree that, at a given moment, individuals on average can represent three to four simple objects worth of information in VSTM in a precise enough format to inform their decisions on what they remember. In other words, when individuals are presented with a visual display that contains more than three to four simple objects worth of information to remember, their VSTM representation of some parts of the display becomes incomplete or too imprecise to make accurate judgments about what they remember.

Furthermore, individuals reliably differ in their VSTM capacity (K. C. Adam, Mance, Fukuda, & Vogel, 2015; Awh, Barton, & Vogel, 2007; Cowan et al., 2005; K. Fukuda, Vogel, Mayr, & Awh, 2010b; K. Fukuda, Woodman, & Vogel, 2015; Shipstead, Harrison, & Engle, 2015); some individuals can represent four or more objects worth of information, while others can represent as little as two or fewer objects worth of information in a precise enough format to inform their decisions about what they remember. Here, we took these reliable individual differences in VSTM capacity to our advantage to test whether VSTM capacity determines the “bandwidth” of VLTM encoding. If VSTM capacity determines the amount of information successfully encoded into VLTM, individuals with high capacity should encode more items at a given time than those with lower capacity. Critically, this relationship should only emerge when the amount of visual information to encode saturated their VSTM (e.g., above Set Size 3 or 4).^1^

## Experiments 1a and 1b: VSTM capacity predicts object VLTM encoding when VSTM is saturated

In Experiments 1a and 1b, we focused on the encoding of a relatively simple form of VLTM—namely, the object VLTM. After measuring individuals’ VSTM capacity, we had participants encode a varying number of pictures of real objects at a time. Subsequently, participants’ VLTM for the encoded pictures were assessed. If VSTM capacity determines the bandwidth of VLTM encoding, we should expect that individual differences in VSTM capacity predict the VLTM performance only when the encoding set size saturated individuals’ VSTM capacity. To examine the effect of encoding intention on VLTM encoding, we ran the same experiment in both an incidental learning condition (i.e., individuals were unaware of the object recognition task; Experiment 1a) and an intentional learning condition (i.e., individuals were informed about the object recognition task prior to the object encoding task; Experiment 1b).

### Method

#### Participants

After signing the consent form approved by the Institutional Review Board, 55 students at the University of Oregon (28 for Experiment 1a and 27 for Experiment 1b) with normal (or corrected-to-normal) vision participated for the introductory psychology course credits.

#### Power calculation

In order to test our key prediction about the effect of VSTM capacity on VLTM encoding, we conducted a repeated-measures ANOVA, with one within-subjects factor of set size and one between-subjects factor of intention for learning. Anticipating that we will obtain a moderate effect size (i.e., *f* = 0.25; J. Cohen, 1988) of set size, the a priori-power calculation with alpha level of 0.05, the statistical power of 0.8, and 0.6 correlation coefficients among the repeated measures, indicated that we would need 24 subjects (Faul, Erdfelder, Lang, & Buchner, 2007). This assures that our sample size was sufficient to detect a moderate size effect with 0.8 statistical power.

As for the correlational analyses, we predicted that there will be a strong correlation (*r* = .6) between individuals’ VSTM capacity and VLTM performance for the objects presented in the supracapacity set size (i.e., Set Size 6). This is because of the causal role that we hypothesized VSTM capacity plays in VLTM encoding. Based on this assumption, we would have needed 19 participants to reliably observe the result with the statistical power of 0.8. This assures that our sample size was sufficient to observe the targeted effect.

For the comparison of the correlational strengths for individuals’ VSTM capacity and VLTM performance across set sizes, we were not able to estimate the sufficient sample size to reliably observe the results for our within-subjects design. However, the power for detecting the difference in correlation strengths increases when two correlations share one variable in common and the correlation between the other variables is available (Steiger, 1980). That is exactly how our experiments were designed.

#### Bayes factor analysis

In addition, to appreciate the statistical significance and nonsignificance of our results, we used JASP software (JASP Team, 2019) and calculated Bayes factor using a default parameter setting (Cauchy prior centered on zero with a scale = 0.707). BF_10_ denotes the odds ratio favoring the alternative hypothesis over the null hypothesis, and BF_01_ denotes the odds ratio favoring the null hypothesis over the alternative hypothesis.

#### Stimuli and procedure

##### Color change detection task

**Fig. 1:**
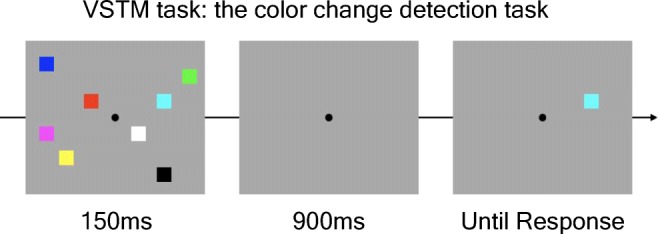
The color change detection task. In this task, an array of colored squares is briefly presented, and participants are asked to hold it in in mind during the retention interval. When a single square is presented, participants indicate if it is the same square as the one that was originally presented in that location. (Color figure online)

A standard color change detection task was administered first to measure individuals’ VSTM capacity (see Fig. 1). In this task, either four or eight colored squares (1.15° × 1.15°) were presented for 150 ms on the screen with a gray background (*memory* array), and individuals were instructed to remember as many of them as possible over a 900-ms retention interval during which the screen remained blank. Then, one colored square was presented at one of the original locations in the memory array (test array), and participants judged if it was the same colored square as the original square presented at that location with a button press (“Z” if they thought it was the same, and “/” if different). The test array remained on the screen until their response. The change frequency was 50% to make sure that any response bias would neither benefit nor penalize their performance. The colors of the memory array were randomly selected from a highly discriminable set of nine colors (red, green, blue, yellow, magenta, cyan, orange, black, and white) without replacement. Participants performed 60 trials each for Set Sizes 4 and 8 conditions in a pseudorandom order.

##### Object encoding task

**Fig. 2:**
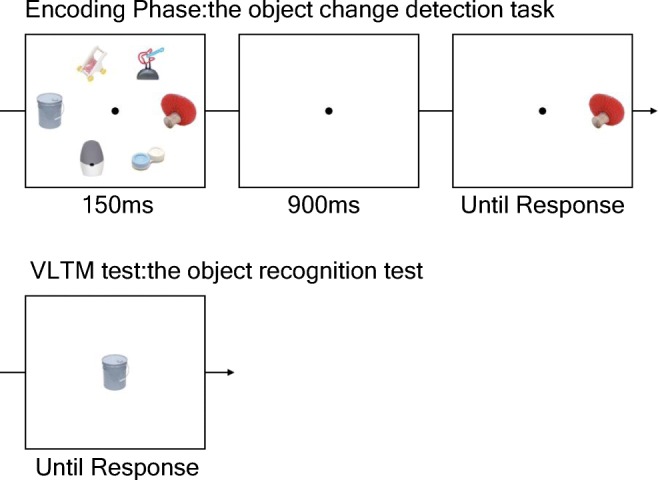
The schematic of Experiments 1a and 1b. The top figure shows the schematic of the encoding phase. In this phase, participants performed an object change detection task. The bottom figure shows the schematic of the object recognition memory test. In this test, participants judged if the picture presented was presented anytime or anywhere during the encoding task. (Color figure online)

Then, participants performed the object encoding task (see Fig. 2). This task was identical to the color change detection task except for two modifications. First, the stimuli presented were pictures of real objects (mean radius = 4.9°) borrowed from Brady and colleagues study (2008), and second, the tested set sizes were two, four, and six. Pictures were selected from a set of 2,400 different pictures without replacement so that none of the pictures appeared on the memory arrays were presented more than once during the encoding task. Participants performed 40 trials each for Set Sizes 2, 4, and 6 in a pseudorandom order.

##### Object recognition task

Following the encoding phase, participants performed the object recognition task (see Fig. 2). In this task, participants were presented with one picture of real objects (mean radius = 4.9°), and they were asked to judge, with a button press, if it was a picture that was presented anytime, anywhere during the encoding phase (“O” for “Old” or studied, and “N” for “New” or never seen). The picture stayed on the screen until their response. Forty previously presented (*old*) pictures for each set size and 120 *new* pictures were tested in a pseudorandom order. Of note, a picture that was tested during the object encoding task was never tested in this task.

### Results

#### Color change detection task

**Fig. 3:**
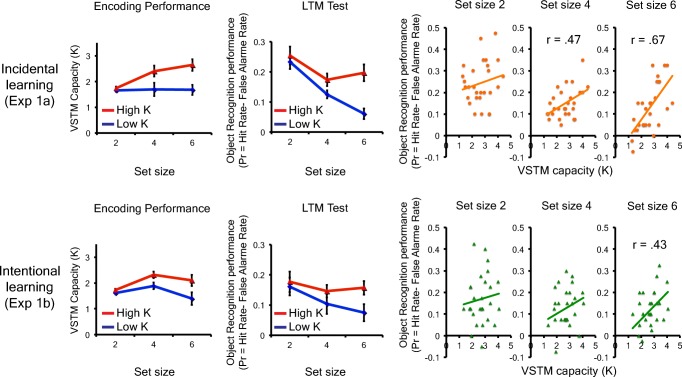
The results of Experiment 1a and 1b. The top row shows the result of Experiment 1a, and the bottom shows the results of Experiment 1b. The left panels show the object change detection performance of high and low capacity (K) groups across set sizes, and the recognition performance of high and low capacity (K) groups across set sizes. The right scatterplots show the correlations between individuals’ visual short-term memory capacity estimate and recognition performance for each condition. The error bars represent the standard error of the mean. (Color figure online)

First of all, individuals’ performance on the color change detection task was converted to VSTM capacity estimate for each set size (K4 for Set Size 4 and K8 for Set Size 8) using a standard formula (Cowan, 2001). K4 and K8 were averaged to compute a single metric for individuals’ VSTM capacity estimates (Kcolor). The mean Kcolor score was 2.6 (*SD* = 0.87) and 2.7 (*SD* = 0.71) for Experiments 1a and 1b, respectively. For a demonstrative purpose, individuals were divided by a median split, into high K (mean K = 3.4, *SD* = 0.52 for Experiment 1a, and mean K = 3.3, *SD* = 0.44 for Experiment 1b) and low K (mean K = 1.9, *SE* = 0.47 for Experiment 1a, and mean K = 2.1, *SE* = 0.40 for Experiment 1b) groups (see Fig. 3).

#### Object encoding task

In the object encoding task, the change detection accuracy for each set size was converted to the capacity estimate. The capacity estimate for each set size was K2 = 1.7 (*SD* = 0.26), K4 = 2.0 (*SD* = 0.94), and K6 = 2.2 (*SD* = 0.92) for Experiment 1a, and K2 = 1.6 (*SD* = 0.21), K4 = 2.1 (*SD* = 0.50), and K6 = 1.7 (*SD* = 1.01), for Experiment 1b (see Fig. 3). The results were analyzed by a repeated-measures ANOVA with two factors (learning intention, set size). As expected, there was a significant set size effect, *F*(2, 106) = 5.85, *p* < .01, η_p_^2^ = 0.1, BF_10_*=* 3.53. In other words, the K estimates increased from Set Size 2 to Set Size 4 and stopped increasing thereafter (as supported by marginally significant linear, *F*(1, 53) = 3.4, *p* = .07, η_p_^2^ = 0.06, and significant quadratic, *F*(1, 51) = 8.9, *p* < .01, η_p_^2^ = 0.14, effects. There was no main effect of learning intention, *F*(1, 53) = 1.1, *ns,* BF_01_*=* 2.71. Furthermore, we examined the correlation between individuals’ VSTM capacity estimated from the color change detection task and from the object encoding task. Here, we found that there was a significant positive correlation between the two estimates for both Experiment 1a (*r* = .65, *p* < .01) and 1b (*r* = .49, *p* < 0.01). This suggests that VSTM capacity estimated by the canonical color change detection task predicted the amount of object representations that participants encoded into their VSTM.

#### Object recognition task

Here, we measured individuals’ corrected recognition performance (Pr = hit rate − false alarm) for each set size. The hit rate was calculated as the proportion of correct responses for *old* trials, and the false alarm was calculated as the proportion of incorrect responses for *new* trials. The results were first analyzed by a repeated measures ANOVA with two factors (learning intention, set size; see Fig. 3). First of all, there was a strong set size effect, *F*(2, 106) = 22.0, *p* < .001, η_p_^2^ = 0.29, BF_10_*=* 8.60×10^5^). In other words, Pr scores decreased from Set Size 2 to Set Size 4 and stopped decreasing thereafter (as supported by both significant linear, *F*(1, 53) = 37.5, *p* < .001, η_p_^2^ = 0.41, and quadratic, *F*(1, 53) = 5.6, *p* < .05, η_p_^2^ = 0.1, effects. There was no main effect of learning intention, *F*(1, 53) = 2.61, *ns*, BF_01_*=* 1.26. Importantly, this set-size-dependent reduction in the recognition accuracy does not necessarily mean that participants encoded a smaller number of visual objects from the display as the set size increased. That is, even if one can remember two objects, regardless of the set size, the likelihood that the encoded objects get tested in the recognition test decreases as the set size increase beyond two. Rather, our results demonstrate that there is a capacity limit in how much information we can encode into VLTM at a given time.

Next, we examined the correlations between individuals’ VSTM capacity and corrected recognition performance. Here, we found that although the correlations between the capacity estimate and the recognition performance was not significant for subcapacity set size (*r* = .19, *ns,* and *r* = .20, *ns,* for Set Size 2 in Experiments 1a and 1b, respectively), they became stronger as set size surpassed their VSTM capacity (*r* = .47, *p* < .01, and *r* = .25, *ns*, for Set Size 4 in Experiments 1a and 1b, respectively; *r* = .67, *p* < .01, and *r* = .43, *p* < .05, for Set Size 6 in Experiments 1a and 1b, respectively). Critically, Steiger’s (1980) *Z* test revealed that the difference in the strength of the correlation with VSTM capacity for subcapacity set size (i.e., Set Size 2) and supracapacity set size (i.e., Set Size 6) was statistically reliable for both experiments. (Steiger’s *Z* test: *Z* = 2.74, *p* < .01 for Experiment 1a with correlation between VLTM for Set Size 2 and Set Size 6 = 0.42; Steiger’s *Z* test: *Z* = 2.17, *p* < .05 for Experiment 1b with correlation between VLTM for Set Size 2 and Set Size 6 = 0.69). Thus, these results confirmed our hypothesis that VSTM capacity predicts the “bandwidth” of VLTM encoding.

### Discussion

In Experiments 1a and 1b, we confirmed the first and the most basic corollary of our “bandwidth” account of VLTM encoding irrespective of the intention for learning. That is, VSTM capacity predicts the amount of VLTM encoded from a display only when the displayed information exceeds individuals’ VSTM capacity. This specificity is critical because it negates the possibility that high capacity individuals were better at any memory tasks because they are those who tend to be more motivated.

## Experiment 2a and 2b: VSTM capacity predicts relational VLTM encoding when VSTM is saturated

To extend our finding to arguably different types of VLTM, we investigated the role of VSTM capacity in creating relational VLTM. Relational memory refers to the memory of interrelations among multiple memory representations, and researchers have argued that it has a specific reliance on the hippocampus and related medial temporal lobe regions (N. J. Cohen, Poldrack, & Eichenbaum, 1997; Davachi, 2006; Davachi & Wagner, 2002; Hannula & Ranganath, 2008; Kumaran & Maguire, 2005; Prince, Daselaar, & Cabeza, 2005; Squire, 1992). The experimental design was very similar to Experiment 1. After the measurement of VSTM capacity, participants proceeded to the relational encoding task followed by a VLTM recognition test. Here, we chose the arrays of colored squares as the relational stimuli because, unlike pictures of real objects, each array is nearly identical in terms of their components (i.e., a selection of squares from nine possible colors), but the difference is determined by the relative positions of the squares (i.e., where is the red square in relation to the blue square?). Therefore, to perform well on the later recognition test, it is critical to have encoded the relational information of the squares. If VSTM capacity also determines the “bandwidth” of VLTM encoding for relational information, individuals’ VSTM capacity should be positively correlated with the VLTM recognition performance only when their VSTM capacity is saturated during encoding (i.e., Set Size 8). Similarly to the previous experiments, we ran two versions of the same studies to test both incidental (Experiment 2a) and intentional (Experiment 2b) learning.

### Method

#### Participants

After signing the consent form approved by the Institutional Review Board, 51 students at the University of Oregon (27 for Experiment 2a, and 24 for Experiment 2b) with normal (or corrected-to-normal) vision participated for the introductory psychology course credits.

#### Power calculation

In order to test our key prediction about the effect of VSTM capacity on the encoding of relational VLTM, we conducted a repeated-measures ANOVA, with one within-subjects factor of set size and one between-subjects factor of intention for learning. Anticipating that we will obtain a moderate effect size (i.e., *f* = 0.25; J. Cohen, 1988) of set size, the priori-power calculation with the same parameter setting as Experiment 1 indicated that we would need 24 subjects (Faul et al., 2007). This assures that our sample size was sufficient to detect a moderate size effect size with 0.8 statistical power.

As for the correlational analyses, we predicted that there will be a strong correlation (*r* = .60) between individuals’ VSTM capacity and VLTM recognition performance for the supracapacity set size arrays (i.e., Set Size 8). Based on this assumption, we would have needed 19 participants to reliably observe the result with the statistical power of 0.8. This assures that our sample size was sufficient to observe the targeted effect.

#### Stimuli and procedure

##### Color change detection task

The task was identical to the ones used in the previous experiments.

##### Relational encoding task

**Fig. 4:**
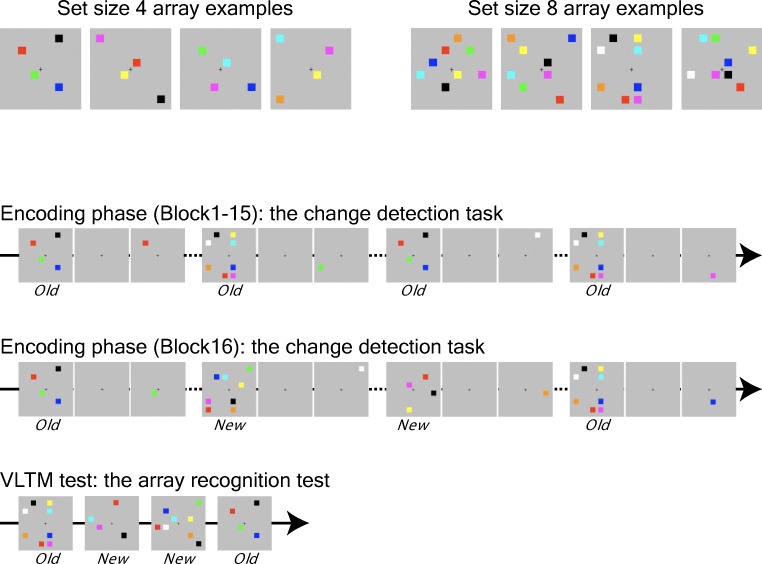
The example stimuli and the schematic of Experiments 2a and 2b. The top panel shows some examples of Set Size 4 and Set Size 8 arrays used in the experiments. The bottom panel shows the schematic of the experiments. In the first 15 blocks of the encoding phase, participants performed the color change detection tasks on 30 arrays (15 each for Set Size 4 and Set Size8) that were repeatedly presented across 15 blocks. *Old* indicates that the array shown above was a repeating array. In the 16th block of the encoding phase, participants performed the color change detection task on the *old* arrays as well as 60 (30 each for Set Size 4 and Set Size 8) *new* arrays that were never presented during the encoding phase so far. In the VLTM test, participants were presented with *old* and a different set of *new* arrays and judged whether they have seen the arrays during the encoding phase. (Color figure online)

Next, participants performed the relational encoding task. The task was identical to the color change detection task except for the following modifications. First, we created 15 Set Size 4 and 15 Set Size 8 arrays that participants encoded repeatedly throughout the encoding phase (*old* arrays). To do so, 15 different spatial layouts were created by selecting eight (Set Size 8) locations on the 6 × 6 grid spanning across the entire visual field. To avoid high similarity amongst spatial layouts for Set Size 4 *old* arrays, the 15 layouts for Set Size 4 arrays were manually created using the same 6 × 6 grid (see Fig. 4). Then, for each layout, a color value was randomly assigned to each location from the set of nine colors without replacement. Participants performed the change detection task on these arrays for 15 blocks. In each block, each *old* array was presented twice in a pseudorandom order. Therefore, by the end of block 15, each *old* array was exposed 30 times. Importantly, the tested location for each *old* array was randomly determined at every exposure. This ensured that the change detection performance is not contaminated by associative learning between each *old* array and its tested location. After block 15, participants performed another block of the change detection task in which they saw 30 *old* arrays (15 Set Size 4 arrays and 15 Set Size 8 arrays) twice and 60 newly created arrays (30 Set Size 4 arrays and 30 Set Size 8 arrays) once in a pseudorandom order. This allowed us to measure the effect of repeated exposures on VSTM capacity while controlling for the general practice benefit.

##### Relational recognition test

After the VLTM encoding task, which lasted for approximately an hour and a half, participants performed the relational recognition task. In this task, participants were presented with one spatial array of colored squares at a time, and they were asked to judge, by a button press, if it was an array that was presented during the encoding phase. The array stayed on the screen until response. Fifteen previously presented *old* arrays for each set size and 15 *new* arrays for each set size were tested in a pseudorandom order.

### Results

#### Color change detection task

**Fig. 5:**
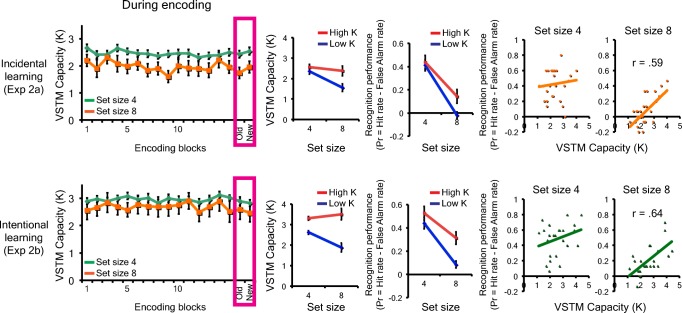
The results of Experiments 2a and 2b. The top row shows the result of Experiment 2a, and the bottom shows the result of Experiment 2b. The left panels show the color change detection performance of high and low capacity (K) groups for Set Sizes 4 and 8 across the relational encoding blocks. *Old* and *new* indicate the performance for the repeated arrays and unrepeated new arrays in the last encoding block. The middle panels show the mean visual short-term memory capacity estimate for each set size across the encoding blocks and the recognition performance of high and low capacity (K) groups for each set size. The right scatterplots show the correlation between individuals’ visual short-term memory capacity estimate and recognition performance for each condition. The error bars represent the standard error of the mean. (Color figure online)

Individuals’ VSTM capacity score (K) was calculated as the average of K estimate for Set Size 4 (mean K4 = 2.6, *SD* = 0.59 for Experiment 2a, and mean K4 = 2.6, *SD* = 0.62 for Experiment 2b) and Set Size 8 (mean K8 = 2.1, *SD* = 1.04 for Experiment 2a, and mean K4 = 2.5, *SD* = 1.34 for Experiment 2b). This resulted in the mean K score of 2.3 (*SD* = 0.69) and 2.6 (*SD* = 0.90) for Experiments 2a and 2b, respectively. The difference in the K scores between experiments did not reach the statistical significance (*p* > .20). For a demonstrative purpose, individuals were divided into high (mean K = 2.9, *SD* = 0.54 and mean K = 3.3, *SD* = 0.64 for Experiments 2a and 2b) and low K (mean K = 1.8, *SD* = 0.31 and mean K = 1.8, *SD* = 0.35 for Experiments 2a and 2b) groups by a median split (see Fig. 5).

#### Relational encoding task

For both experiments, individuals were presented with each *old* array 30 times across the span of the encoding task. To test the learning effect on the change detection performance, we tested if there was an improvement in performance over time (see Fig. 5). Although there was a reliable set size effect, *F*(1, 49) = 12.79, *p* < .01, η_p_^2^ = 0.21 for K4 > K8 (BF_10_*=* 3.15×10^12^) and a reliable effect of learning intention, *F*(1, 49) = 9.22, *p* < .01, η_p_^2^ = 0.16 for intentional learning > incidental learning (BF_10_*=* 9.43), there was no interpretable effect of repetition, *F*(14, 686) = 0.74, *ns* (BF_01_*=* 2.68×10^4^). In other words, the capacity estimates for each set size remained constant across repetitions for both set sizes in both experiments. There was no interpretable interaction among the three factors (*p*s > 0.32, BF_01_ > 1.2)

To further test the effect of repetition on VSTM capacity estimate, the K scores for *old* arrays and *new* arrays in the last block of the encoding task were compared (see Fig. 5). A repeated-measures ANOVA with three factors of learning intention, set size, and array repetition revealed a significant set size effect, *F*(1, 49) = 13.2, *p* < .01, η_p_^2^ = 0.21 (BF_10_*=* 1.10×10^3^). In addition, there was a significant effect of learning intention, *F*(1, 49) = 4.55, *p* < .05, η_p_^2^ = 0.09 (BF_10_*=* 1.86). There was no effect of array repetition, *F*(1, 49) = 0.10, *ns* (BF_01_*=* 6.21) or interpretable interactions among the three factors (*p*s > 0.16, BF_01_ > 1.8). In addition, strong correlations between the K estimates for *old* and *new* arrays (*r*s > .70, *p*s < .01) revealed that the individual differences were also preserved even after repeated exposures to the arrays. This is consistent with previous observation by Olson and colleagues (Olson, Jiang, & Moore, 2005).

##### Relational recognition test

First, to examine the difference in distinctiveness for Set Size 4 and Set Size 8 arrays, we compared the false-alarm rates for Set Size 4 and Set Size 8 arrays. If one type of array was less distinctive than the other, the new arrays of less distinctive set size would be more likely to be confused as old than would those of more distinctive set size. The paired *t* tests revealed that the false-alarm rates for Set Size 4 and Set Size 8 arrays were somewhat different in Experiment 2a, but not in 2b, *t*(26) = 2.01, *p* = .06, BF_01_ = 0.87 for Experiment 2a; *t*(23) = 0.83, *ns*, BF_01_ = 3.41 for Experiment 2b. These results showed that our manipulation was somewhat successful in reducing the differences in the distinctiveness between Set Size 4 and Set Size 8 arrays.

Next, to assess the relational VLTM, we measured the corrected recognition performance (Pr = hit rate − false alarm). A repeated-measures ANOVA with two factors (instruction, set size) revealed the following results (see Fig. 5). First, there was a main effect of set size, *F*(1, 49) = 97.36, *p* < .001, η_p_^2^ = 0.67, BF_10_*=* 1.99×10^12^, that Pr scores for Set Size 4 arrays were larger than that for Set Size 8 arrays. Although there was a main effect of learning intention, *F*(1, 49) = 4.78, *p* < .05, η_p_^2^ = 0.09, BF_10_*=* 0.88, there was no significant interaction between learning intention and set size, *F*(1, 49) = 1.34, *ns*, BF_01_ = 1.99.

Next, we examined the correlation between VSTM capacity and VLTM recognition performance. Here, we found that individuals’ K scores did not significantly correlate with Pr scores for Set Size 4 (*r* = .10, *ns*, and *r* = .28, *ns*, for Experiments 2a and 2b, respectively), but they did with those for Set Size 8 (*r* = .59, *p* < .01, and *r* = .64, *p* < .01 for Experiments 2a and 2b, respectively). Critically, Steiger’s (1980) *Z* test revealed that the difference in the strength of the correlation with VSTM capacity for Set Size 4 and Set Size 8 was statistically reliable for both experiments (Steiger’s *Z* test: *Z* = 2.03, *p* < .05 for Experiment 2a with correlation between VLTM for Set Size 4 and Set Size 8 = 0.07; Steiger’s *Z* test: *Z* = 2.15*, p* < .05 for Experiment 2b with correlation between VLTM for Set Size 4 and Set Size 8 = 0.56). Thus, these results confirmed our hypothesis that VSTM capacity predicts the “bandwidth” of VLTM encoding only when the information to encode surpasses one’s VSTM capacity.

### Discussion

The results demonstrated that VSTM capacity also predicted the encoding of relational VLTM only when individuals’ VSTM capacity was saturated. The finding was consistent regardless of participants’ intention for learning. Together with the results of Experiments 1a and 1b, our results demonstrated that VSTM capacity predicts the “bandwidth” for VLTM encoding regardless of the type of information (i.e., object LTM or relational LTM) and the subjects’ intention for learning (i.e., incidental learning or intentional learning).

#### Determining the locus of the “bandwidth” of VLTM encoding

Across all the experiments, we consistently observed that the amount of information represented in VSTM predicted the amount of information encoded into VLTM—namely, the “bandwidth” of VLTM encoding. One critical caveat, however, is that our evidence so far is all correlational. Given that individual differences in VSTM capacity predict a variety of higher cognitive functions (e.g., Cowan et al., 2005; Cowan, Fristoe, Elliott, Brunner, & Saults, 2006; K. Fukuda, Vogel, et al., 2010b; K. Fukuda et al., 2015; Shipstead et al., 2015; Shipstead, Redick, Hicks, & Engle, 2012; Unsworth, Fukuda, Awh, & Vogel, 2014), demonstrating that a positive correlation between VSTM capacity and VLTM encoding is not sufficient to claim a causal link between them. Therefore, to gain more direct evidence for our account, we conducted additional experiments in which we causally manipulated the dissociable aspects of VSTM and examined their impact on VLTM encoding.

More precisely, our VLTM encoding tasks involved three dissociable VSTM processes. First, visual information had to be encoded into VSTM. Second, the encoded information had to be maintained in VSTM across the retention interval. Third, the maintained representation had to be evaluated to select an appropriate response for the task at hand (e.g., change detection task). At this point, it is unclear whether these three processes are directly involved in VLTM encoding.

For instance, researchers have theorized that the act of active maintenance (Atkinson & Shiffrin, 1971; Khader, Ranganath, Seemuller, & Rosler, 2007; Ranganath, Cohen, & Brozinsky, 2005; Rundus, 1971) determines the encoding success. In other words, VSTM serves as the incubator for information to become VLTM representations. Although this view has received both support and criticism, it is plausible that VSTM maintenance has a direct contribution to VLTM encoding.

Alternatively, the evaluation of maintained VSTM representations could have contributed to VLTM encoding. In our experimental designs so far, VSTM representations were always evaluated at the end of each encoding trial (e.g., compared against the test item), and thus it is also plausible that VLTM was created at this stage of VSTM processes. Thus, in the next experiments, we decided to manipulate each VSTM processes to examine their roles on VLTM encoding.

## Experiment 3a: VSTM maintenance and evaluation do not contribute to VLTM encoding

To determine the effect of VSTM encoding, maintenance and evaluation on VLTM encoding, we orthogonally manipulated the number of encoding opportunities, the maintenance duration, and the necessity of the evaluation in the VLTM encoding task. If the number of VSTM encoding opportunities is the key for VLTM encoding, then the stimuli that were encoded more times should be better remembered than those that were not. On the other hand, if the duration of VSTM maintenance is the key, then the stimuli that were maintained longer should be better remembered than those that were not. Alternatively, if it is the evaluation of VSTM representation, then the stimuli whose VSTM representations were evaluated should be better remembered than those that were not.

### Method

#### Participants

After signing the consent form approved by Institutional Review Board, 23 students with normal (or corrected-to-normal) vision at the University of Oregon participated for the introductory psychology course credits.

#### Power calculation

In order to test our key prediction about the role of VSTM maintenance on VLTM encoding, we conducted a repeated-measures ANOVA, with one within-subjects factor of encoding condition (i.e., *base*, *base3*, *short3* and *long*). Anticipating that we will obtain a moderate effect size (i.e., *f* = 0.25; J. Cohen, 1988) of encoding condition, a priori-power calculation with the same parameter setting as Experiment 1 indicated that we would need 19 subjects (Faul et al., 2007). This assures that our sample size was sufficient to detect a moderate size effect size with 0.8 statistical power.

#### Procedure

##### Object encoding task

**Fig. 6:**
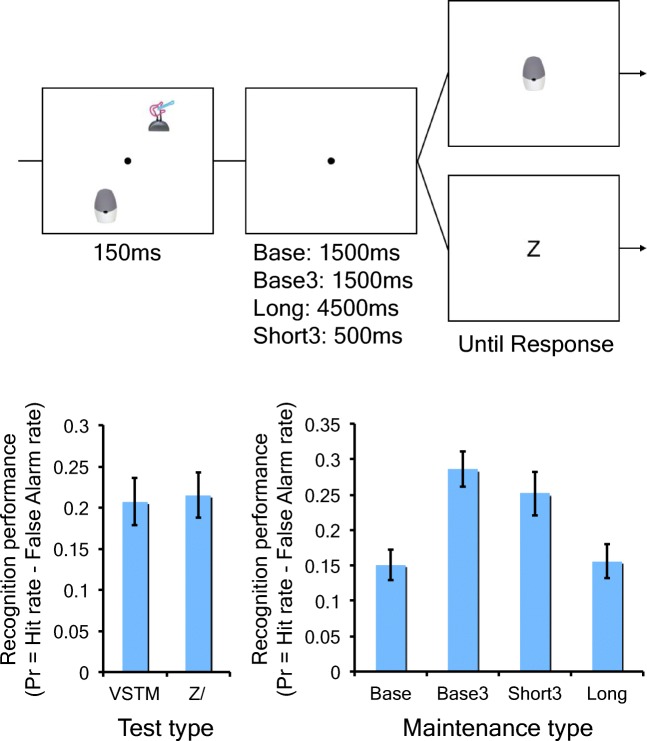
The schematic and the results of Experiment 3a. The top panel shows the schematic of the object encoding task. The bottom right panel shows the recognition performance for two test types. The bottom left panel shows the recognition performance for four maintenance conditions. The error bars represent the standard error of the mean

Participants performed a modified version of the object change detection task used in Experiments 1a and 1b (see Fig. 6). In this task, every trial presented two pictures of real objects for 150 ms, and participants were asked to remember them across the retention interval. In the *base* condition, the retention interval was 1.5 seconds long, and the pictures were presented only once throughout the encoding phase. In the *base3* condition, each trial had a 1.5-second-long retention interval, but each picture was encountered three times over the course of the experiment to ensure that pictures in this condition were encoded into VSTM three times. In the *long* condition, the retention interval was three times longer (4.5 seconds) than that of *base* condition to equate the total duration of the VSTM maintenance with the *base3* condition. In the *short3* condition, the retention interval was a third in duration (.5 second) in comparison to the *base* condition, but each trial was encountered three times across the experiment to equate the total duration of VSTM maintenance with the *base* condition.

Orthogonal to the retention interval manipulation, the type of the test was also manipulated. In one half of trials in each condition, the retention interval was followed by a typical VSTM test, in which, participants had to judge if the picture presented at the center was identical to the pictures presented in the preceding memory array (*VSTM test* condition). The change frequency was 50% to control for any response bias. On the other trials (*Z/ test* condition), the retention interval was followed by the presentation of “z” or “/” at the center of the screen. Here, participants were asked to simply press the corresponding key on the keyboard.

The number of encoding trials was 60 for the *base* and *long* conditions, and 180 for the *base3* and *short3* conditions to ensure that the same number of pictures would be tested in the following VLTM recognition test. Importantly in *VSTM test* condition, the same picture was tested across multiple exposures in order to leave one picture untested for the VLTM recognition test. The encoding phase lasted for approximately 1.5 hours.

##### Object recognition test

The VLTM recognition test was identical to the one used in Experiment 1a and 1b. Thirty *old* pictures for each condition, 30 × 4 (*base*, *base3*, *long* and *short3*) × 2 (*VSTM test* and *Z/ test*) = 240 pictures in total, and 60 *new* pictures were presented during the test. Of note, none of the pictures presented as a test item in the *VSTM test* condition were presented.

### Result

#### Object encoding task

The performance on the encoding task was analyzed for each condition. For *Z/ test* conditions, not surprisingly, the accuracy was at ceiling across all conditions (accuracies > .98 for *base*, *base3*, *short3*, and *long*), *F*(3, 66) = 1.58, *ns*, BF_01_*=* 2.88. For *VSTM test* conditions, there was a small but reliable effect of the VSTM maintenance duration such that the accuracy for the *short3* condition (mean = 0.94, *SD* = 0.04) was better than those for the *base* and *base3* conditions (mean = 0.91, *SD* = 0.08 for *base*; mean = 0.92, *SD* = 0.07, for *base3*), *t*(23) > 2.27, *p*s < .05, BF_10_ > 1.82, which were all better than that for *long* condition (mean = 0.84, *SD* = 0.09), *t*(23) > 4.69, *p* < .01, BF_10_ = 2.44×10^2^. This resulted in a small but detectable reduction in the number objects maintained in VSTM as the retention interval got longer (K = 1.8, 1,7, 1.6, and 1.4 for *short3*, *base3*, *base*, and *long*, respectively). Although there was a small difference in VSTM performance across retention intervals, we believe that this effect of maintenance duration is fairly limited considering the magnitude of our manipulation (1.5 seconds vs. 4.5 seconds) and previous findings (Naveh-Benjamin & Jonides, 1984).

#### Object recognition test

First of all, the effect of the test was analyzed by averaging across all *base*, *base3*, *short3* and *long* conditions. Markedly, there was no difference in recognition performance based on the type of the test (mean Pr = 0.22, *SD* = 0.10 for *Z/ test* condition, mean Pr = 0.21, *SD* = 0.10 for *VSTM test* condition), *t*(23) = 0.73, *ns,* BF_01_ = 3.60 (see Fig. 6). This, together with the recent findings by Schurgin and Flombaum (2018, Experiment 7), clearly demonstrated that the link between VSTM and VLTM encoding was not mediated by the evaluation of VSTM representation. Next, the effect of VSTM maintenance was analyzed. Given the null effect of the test types, the Pr scores were averaged across the test types for the later analyses. A repeated-measures ANOVA revealed that there was a significant effect of learning conditions, *F*(3, 66) = 24.3, *p* < .001, η_p_^2^ = 0.53, BF_10_ = 7.95×10^7^ (see Fig. 6). To further characterize the results, a series of planned *t* tests were conducted. First, as expected, the Pr scores for the *base3* (mean Pr = 0.29, *SD* = 0.12) condition were significantly better than those for the *base* condition (mean Pr = 0.15, *SD* = 0.09), *t*(22) = 5.7, Cohen’s *d =* 1.19, *p* < .001, BF_10_*=* 2.14×10^3^. Strikingly, the Pr scores for the *long* condition (mean Pr = .16, *SD* = 0.11) were statistically equivalent to those for the *base* condition, *t*(22) = 0.28, *ns*, Cohen’s *d =* 0.06*, BF*_*01*_*=* 4.40, and significantly lower than those for the *base3* condition, *t*(22) = 7.4, *p* < .001, Cohen’s *d =* 1.55, BF_10_*=* 7.94×10^4^. On the other hand, the Pr scores for the *short3* condition (mean Pr = 0.26, *SD* = 0.13) was significantly better than those for *base* condition, *t*(22) = 4.6, Cohen’s *d =* 0.95, *p* < .001, BF_10_ = 1.89×10^2^, and statistically equivalent to those for the *base3* condition, *t*(22) = 2.0, *p* = .06, Cohen’s *d =* 0.41, BF_01_*=* 0.88. These results clearly point to the fact that longer VSTM maintenance did not positively impact VLTM encoding.

### Discussion

In Experiment 3a, we directly tested what VSTM processes govern the “bandwidth” of VLTM encoding. Here, we observed no evidence that VSTM evaluation or VSTM maintenance affected VLTM encoding. Taken together, postencoding VSTM processes had a negligible effect on VLTM encoding. As a result, it seems that it was the number of VSTM encoding opportunities that had a significant impact on VLTM encoding.

## Experiment 3b: VSTM encoding governs VLTM encoding

Experiment 3a suggested that postencoding VSTM processes have no direct influence on VLTM encoding. This effectively leaves one process, namely VSTM encoding, as a candidate that governs VLTM encoding. Although this account explains our findings that more encoding opportunities led to better VLTM encoding, it is not the only explanation. One alternative explanation is that when the same stimulus is presented again during the encoding task, it provides an opportunity for participants to retrieve the stimulus from their VLTM, and this incidental retrieval may improve VLTM. Another alternative explanation may be that it is the repeated perceptual exposures, rather than VSTM encoding, that lead to better VLTM encoding. In order to rule out these alternatives, we parametrically manipulated the quality of VSTM encoding through a postperceptual masking procedure while fixing the number of encoding opportunities constant. If VSTM encoding determines VLTM encoding, postperceptual interruption of VSTM encoding should lead to an analogous effect on VLTM encoding. More precisely, we presented masks at various ISIs from the offset of the stimuli. It is established that postperceptual masks disrupt VSTM encoding at various degrees depending on the stimuli to mask ISIs such that the shorter the ISI, the more disruption there is to VSTM encoding (Vogel, Woodman, & Luck, 2006). If VSTM encoding governs VLTM encoding, we should expect that the parametric postperceptual disruption of VSTM encoding leads to the analogous parametric disruption of VLTM encoding. In other words, when we control for the well-known difference in memory strengths between VSTM and VLTM (e.g., Brady, Konkle, Gill, Oliva, & Alvarez, 2013; Schurgin, 2018; Schurgin & Flombaum, 2018), there should be no interaction between the memory types and the effect of stimulus-to-mask ISIs.

### Method

#### Participants

After signing the consent form approved by the Institutional Review Board, 26 students with normal (or corrected-to-normal) vision at the University of Oregon participated for the introductory psychology course credits.

#### Normalization of memory performance

We quantified the VSTM and VLTM performance using the same corrected recognition performance, namely the Pr (i.e., hit rate − false alarm). More precisely, for VSTM performance, the hit rate was defined as the proportion of correct responses for *different* trials and the false-alarm rate was defined as the proportion of incorrect responses for *same* trials. Of note, the calculated Pr values are mathematically equivalent when they are calculated by defining the hit rate as the proportion of correct responses for *same* trials and the false-alarm rate as the proportion of incorrect responses for *different* trials. For VLTM performance, we calculated the Pr in the same way as the previous experiments by defining the hit rate as the proportion of correct trials for *old* condition and the false-alarm rate as the proportion of incorrect trials for *new* condition.

Furthermore, to control for a substantial difference in memory strength between two types of memories (e.g., Brady et al., 2013; Schurgin, 2018; Schurgin & Flombaum, 2018), we normalized the difference in Pr scores between masking and no mask conditions (i.e., Pr difference) in a memory-specific manner. That is, for each memory type, we calculated the relative Pr difference for each masking condition by calculating the proportion score in the range defined by the smallest and largest Pr differences across all masking conditions. In other words, the normalized Pr difference was calculated using the following formula: normalized Pr difference = (Pr difference − min(Pr differences)/(max(Pr differences) − min(Pr differences)) where min(Pr differences) represents the smallest Pr difference across all *masking* conditions and max(Pr differences) represents the largest Pr differences across all *masking* conditions. This normalization procedure allowed us to directly compare the effect of postperceptual masking while controlling for the substantial difference in memory strengths between VSTM and VLTM.

#### Power calculation

First, in order to verify that our postperceptual masking manipulation worked, we conducted a repeated-measures ANOVA, with one within-subjects factor of stimulus-to-mask ISIs (i.e., no mask, 0 ms, 100 ms, 300 ms, 600 ms, and 1,500 ms) on VSTM Pr scores. Anticipating that we will obtain a moderate effect size (i.e., *f* = 0.25; J. Cohen, 1988), a priori-power calculation with the same parameter setting as Experiment 1 indicated that we would need 15 subjects (Faul et al., 2007).

Next, to examine the effect of stimulus-to-mask ISIs on VLTM encoding, we conducted a repeated-measures ANOVA, with one within-subjects factor of stimulus-to-mask ISIs (i.e., no mask, 0 ms, 100 ms, 300 ms, 600 ms, and 1,500 ms) on VLTM Pr scores. Anticipating that we will obtain a moderate effect size (i.e., *f* = 0.25; J. Cohen, 1988), a priori-power calculation indicated that we would need 15 subjects (Faul et al., 2007).

Lastly, in order to test our key prediction, we conducted a repeated-measures ANOVA on normalized Pr differences with two within-subjects factors of memory types (VSTM and VLTM) and stimulus-to-mask ISIs (i.e., 0 ms, 100 ms, 300 ms, 600 ms, and 1,500 ms). Anticipating that we will obtain a moderate effect size (i.e., *f* = 0.25; J. Cohen, 1988), a priori-power calculation indicated that we would need 18 subjects (Faul et al., 2007). Taken together, our sample size was sufficient to detect a moderate size effect size with 0.8 statistical power.

#### Stimuli and procedure

##### Object encoding task

**Fig. 7:**
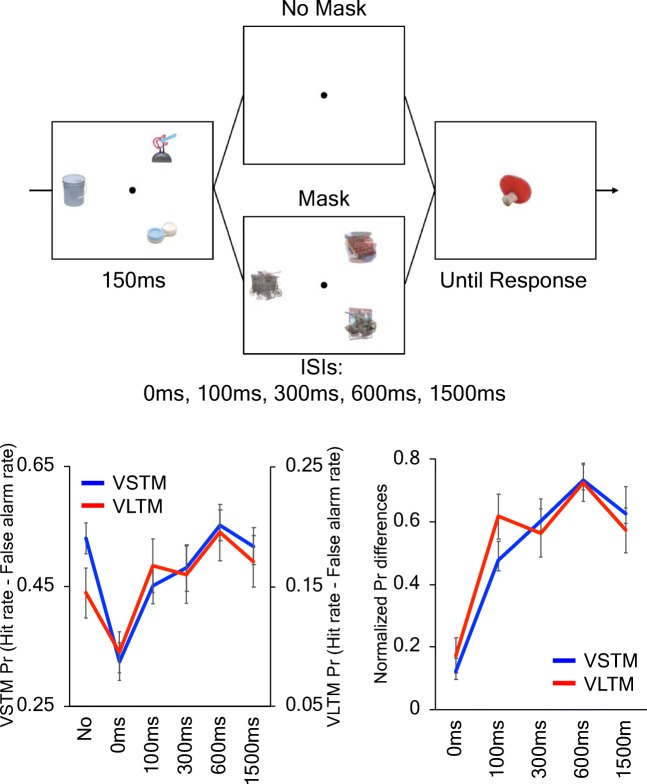
The object encoding task and the results from Experiment 3b. The top panel shows the schematic of the object encoding task. The bottom panels show the VSTM and VLTM performance (Pr scores on the left and normalized Pr differences on the right). The error bars represent the standard error of the mean. (Color figure online)

Participants performed a modified version of the object change detection task used in Experiments 1a and 1b (see Fig. 7). In this task, every trial presented three pictures of real objects for 150 ms, and participants were asked to remember them across the retention interval that was followed by the same VSTM test as in Experiment 3a. In 200 out of 280 trials, short rapid serial presentations of mask stimuli (50 ms each for three stimuli) were presented at each memory item location during the retention interval at 0 ms, 100 ms, 300 ms, 600 ms, or 1,500 ms after the offset of the memory array with equal probabilities. The mask stimuli were created by overlaying multiple pictures not used in the entire experiment. The duration of the retention interval was 1,500 ms for the 0-ms, 100-ms, 300-ms, and 600-ms mask conditions. For the 1,500-ms mask condition, it was set to 2,400 ms to provide enough temporal separation between the mask presentation and the test stimulus.

In the rest of the trials, no mask was presented during the retention interval. To have an equal duration of the retention interval for all masking conditions, one half of the trials had a 1,500-ms-long retention interval, and the other half had a 2,400-ms-long interval. This encoding task lasted for approximately an hour.

##### Object recognition test

The VLTM recognition test was identical to the one used in Experiments 1a and 1b. Forty *old* pictures for each condition (280 total) and 40 *new* pictures were presented during the test. Of note, none of the pictures used as the test items in the object encoding task were presented.

### Result

First, we verified that VSTM and VLTM differed substantially in memory strength by comparing their Pr scores for *no mask* conditions. The comparison revealed a significant effect of memory types such that individuals were much better in VSTM performance than in VLTM performance (mean Pr = 0.53, *SD* = 0.13 for VSTM; mean Pr = 0.15, *SD* = 0.10 for VLTM), *t*(25) = 12.1 *p* < .001, BF_10_*=* 1.54×10^9^. This expected finding is consistent with recent findings (Brady et al., 2013; Schurgin, 2018; Schurgin & Flombaum, 2018), and thus necessitated the memory-specific normalization of memory performances to properly compare the effect of postperceptual masks.

Next, we verified that our postperceptual masks were effective in disrupting VSTM encoding in a parametric manner. A repeated-measures ANOVA revealed that there was a significant main effect of stimulus-to-mask ISI on Pr scores, *F*(5, 125) = 10.4, *p* < .001, η_p_^2^ = 0.29, BF_10_*=* 7.75×10^5^,(see Fig. 7) such that mask-induced disruption monotonically decreased as the stimulus-to-mask ISI got longer. Planned *t* tests revealed that the Pr score was statistically worse than *no mask* condition at 0 ms and 100 ms ISI conditions, *t*(25) = −7.78, *p* < .01, Cohen’s *d* = 1.53, BF_10_ = 3.66×10^5^; *t*(25) = −2.31, *p* < .05, Cohen’s *d* = 0.45, BF_10_ = 1.92 for 0 ms and 100 ms ISI, respectively, and it became no worse than the no mask condition afterwards, *t*(25) = −1.63, *ns*, Cohen’s *d* = 0.32, BF_01_ = 1.51; *t*(25) = 0.66, *ns,* Cohen’s *d* = 0.13, BF_01_ = 3.95; *t*(25) = −0.40, *ns,* Cohen’s *d* = 0.08, BF_01_ = 4.48 for 300 ms, 600 ms, and 1,500 ms ISI, respectively). Using a conventional Bayes factor cutoff of 3 (Jeffreys, 1961), this suggests that VSTM encoding was likely complete by 600 ms after the offset of stimulus.

Next, we examined the effect of postperceptual masks on VLTM encoding. A repeated-measures ANOVA revealed that there was a significant main effect of stimulus-to-mask ISI on Pr differences, *F*(5, 125) = 5.71, *p* < .01, η_p_^2^ = 0.19, BF_10_= 2.53×10^2^ (see Fig. 7) such that mask-induced disruption monotonically decreased as the stimulus-to-mask ISI got longer. Planned *t* tests revealed that the Pr score was statistically worse at 0 ms ISI conditions, *t*(25) = −2.58, Cohen’s *d* = 0.51, *p* < .01, BF_10_ = 3.13 for 0-ms ISI, and it became no worse, if not better, than the no mask condition afterwards, *t*(25) = 1.16, *ns*, Cohen’s *d* = 0.23, BF_01_ = 2.65; *t*(25) = 0.83, *ns*, Cohen’s *d* = 0.16, BF_01_ = 3.52; *t*(25) = 2.22, *p <* .05, Cohen’s *d* = 0.44, BF_10_ = 1.65; *t*(25) = 1.37, *ns,* Cohen’s *d* = 0.27, BF_01_ = 2.09 for 100 ms, 300 ms, 600 ms, and 1,500 ms ISI, respectively. This slight benefit of postperceptual masks presented after the completion of VSTM encoding (i.e., 600-ms ISI) is consistent with the idea that the presence of the postperceptual masks encouraged individuals to “refresh” their VSTM representations, and thus led to better VLTM encoding (Johnson, Reeder, Raye, & Mitchell, 2002).

Lastly, we directly compared the effect of postperceptual masks across two memory types using the normalized Pr differences. As predicted, a repeated-measures ANOVA revealed that there was a significant effect of stimulus-to-mask ISI, *F*(4, 100) = 16.5, *p* < .001, η_p_^2^ = 0.40, BF_10_ = 1.93×10^13^. However, there was no main effect of memory types, *F*(1, 25) = 0.5, *ns*, η_p_^2^ = 0.0, BF_01_ = 6.71, or an interaction between memory types and stimulus-to-mask ISI, *F*(4, 100) = 0.8, *ns*, η_p_^2^ = 0.0, BF_01_ = 12.7. These results, particularly the large Bayes factor in favor of the null result, provide strong evidence for the lack of the interaction between memory types and stimulus-to-mask ISIs, and thus are in line with our hypothesis that postperceptual disruption of VSTM encoding translates to VLTM encoding.

### Discussion

In Experiment 3b, we directly manipulated VSTM encoding to test its impact on VLTM encoding. As predicted, we successfully modulated VLTM encoding by disrupting VSTM encoding. Taken together with the results from Experiment 3a, VSTM encoding governs the encoding of VLTM.

## General discussion

In this study, we attempted to fill in an overlooked hole in our theories of VLTM encoding by characterizing its initial bottleneck. More specifically, we hypothesized that VSTM capacity determines the encoding bandwidth, or the amount of visual information that can be encoded at a given moment, of VLTM. By taking advantage of reliable individual differences in VSTM capacity, we demonstrated that individuals’ VSTM capacity predicted the bandwidth for VLTM encoding regardless of the type of visual information (i.e., object memory or relational memory) and learning intention only when their VSTM is saturated. Furthermore, we causally manipulated the dissociable VSTM processes and found that post-VSTM-encoding processes had a negligible impact on VLTM encoding. Instead, postperceptual disruption of VSTM encoding had a directly translative impact on VLTM encoding. Taken together, our results provided converging evidence that VSTM capacity determines the “bandwidth” of VLTM encoding via the shared encoding bottleneck.

### Alternative Hypothesis 1: Motivation rather than VSTM capacity?

Although we have demonstrated that VLTM encoding can be causally manipulated through direct manipulations of VSTM processes, the correlational evidence for the link between VSTM capacity and VLTM encoding in Experiments 1 and 2 might raise a concern that what underlies these correlations are the individual differences in intrinsic motivation to perform the task well. More precisely, one could argue that those with higher intrinsic motivation might perform any memory tasks better than those with lower motivation. This alternative explanation is certainly compatible with the reliable positive correlations between individuals’ VSTM capacity and VLTM recognition performance for supracapacity set sizes. However, this alternative account cannot explain why such a correlation was not observed for the recognition performance for subcapacity set size even though the recognition performance was clearly below ceiling. This, in turn, suggests that the robust relationship between VSTM capacity and VLTM encoding only emerges when individuals’ VSTM is saturated. Although it is still possible that low capacity individuals selectively lowered their motivation when presented with supracapacity set sizes, our results demonstrated that the performance for VSTM and VLTM tasks were affected in parallel. This is consistent with our “bandwidth” account of VLTM encoding.

### Alternative Hypothesis 2: Systematic differences in stimulus properties and encoding strategies?

Other important factors that could influence the observed pattern of memory performance include systematic variations in stimulus properties and individual-specific encoding strategies. Indeed, recent studies have demonstrated that some stimuli are more memorable than others for both stimulus-specific and context-specific reasons (e.g., Bainbridge, Dilks, & Oliva, 2017; Bainbridge, Isola, & Oliva, 2013; Borkin et al., 2016; Bylinskii, Isola, Bainbridge, Torralba, & Oliva, 2015; Konkle, Brady, Alvarez, & Oliva, 2010). To avoid such contamination, we have assigned our stimuli randomly across all encoding (e.g., set sizes, number of repetitions, masking) and testing (e.g., old and new) conditions in each experiment. This made it unlikely that the stimuli in a specific encoding condition were more memorable than those in other conditions. Thus, our results are not likely driven by systematic variations in the stimulus properties that determine their memorability.

Next, it is also plausible that individuals utilized various encoding strategies that have different implications for VLTM performance. For example, it is well established that semantic encoding strategies benefit LTM more than perceptual encoding strategies (e.g., Craik, 1983; Craik & Lockhart, 1972; Craik & Tulving, 1975; Craik & Watkins, 1973; Fisher & Craik, 1977; Moscovitch & Craik, 1976), and it is possible that some individuals (i.e., high VSTM capacity individuals) engaged more in semantic encoding strategies than the others do (i.e., low VSTM capacity individuals). This alternative would predict that high capacity individuals show superior VLTM performance irrespective of encoding set sizes. However, that is not what we found. What we found instead was that high capacity individuals outperformed low capacity individuals only when their VSTM capacity was saturated, and thus this pattern of the results is most compatible with our hypothesis that VSTM encoding governs the “bandwidth” of VLTM encoding.

### Alternative Hypothesis 3: Attentional capacity rather than VSTM capacity?

VSTM capacity measures are heavily influenced by individuals’ ability to regulate what gets encoded into their limited mental workspace (K. Fukuda, Awh, et al., 2010a; K. Fukuda & Vogel, 2009, 2011; K. Fukuda et al., 2015; Liesefeld, Liesefeld, & Zimmer, 2014; Linke, Vicente-Grabovetsky, Mitchell, & Cusack, 2011; McNab & Klingberg, 2008, Unsworth et al., 2014; Vogel, McCollough, & Machizawa, 2005). Does this mean that the encoding “bandwidth” for VLTM encoding is set by individuals’ attentional capacity instead? Our results are consistent with this account to a certain extent. In fact, it is our view that individuals’ ability to attentionally regulate what gets VSTM explains a major portion of the individual differences in VSTM capacity (K. C. Adam et al., 2015; K. Fukuda, Awh, et al., 2010a; K. Fukuda et al., 2015; Unsworth et al., 2014). Importantly, however, our results in Experiment 3b demonstrated that it is not the attentional allocation to perceptually available stimuli but rather the successful encoding into VSTM that determines VLTM encoding. More precisely in this experiment, we parametrically interrupted VSTM encoding after stimuli were perceptually attended for a fixed amount of time. If the attentional allocation to the perceptually available stimuli was sufficient to create VLTM representations, the parametric postperceptual disruption of VSTM encoding should not have affected VLTM encoding. However, we found that the degree of postperceptual disruption of VSTM encoding transferred to VLTM encoding. This indicates that the attentional allocation to perceptually available stimuli is not sufficient to explain the pattern of our results. Instead, it is the VSTM encoding that continues on postperceptually that contributes to VLTM encoding. Of note, some theories state that this VSTM process is subserved by internally oriented attention (e.g., Chun, Golomb, & Turk-Browne, 2011), and in that sense, our results demonstrate that a particular aspect of attention is directly involved in the encoding of both VSTM and VLTM.

### Alternative Hypothesis 4: Distinct rather than shared encoding processes between VSTM and VLTM?

Another plausible interpretation of our results is that VSTM and VLTM representations are encoded separately by dissociable mechanisms that are equally disrupted by postperceptual masking. Although our “bandwidth” account provides a more parsimonious explanation without necessitating multiple encoding mechanisms, our current data alone cannot rule out this alternative account. Interestingly, however, Brady et al. (2013) found that the fidelity of VLTM representations is set by the fidelity of VSTM representations, thus demonstrating another parallel between VSTM and VLTM. Together with this finding, the most parsimonious interpretation of our results is that VSTM encoding governs the “bandwidth” of VLTM encoding.

On the other hand, our data are in line with a current theoretical perspective that STM is an embedded process that resides in LTM. More precisely, STM is not a set of distinct mental operations but rather is an activated portion of LTM (Cowan, 2001; K. Fukuda & Woodman, 2017; Jonides et al., 2008; Nairne, 2002; Ruchkin, Grafman, Cameron, & Berndt, 2003). In other words, VSTM capacity is conceptualized as the amount of VLTM that can be activated at a given time. Our findings contribute to this theoretical framework by demonstrating that the bandwidth at which one can encode new VLTM is explained by one’s capacity to actively represent already-encoded VLTM.

### Implications and future directions

Our “bandwidth” account offers a plausible explanation for the robust relationship between fluid intelligence (gF) and crystallized intelligence, namely the accumulated LTM. For many years, fluid intelligence has been theorized to determine the rate of acquisition of crystallized intelligence (Cattell, 1957, 1963; Schweizer & Koch, 2001). However, its specific mechanisms have yet to be identified. Given the tight relationship between VSTM capacity and fluid intelligence (Cowan et al., 2005; K. Fukuda, Vogel, et al., 2010b; Shipstead et al., 2012; Unsworth et al., 2014), our “bandwidth” account proposes a plausible mechanism that fluid intelligence explains the acquisition rate of crystallized intelligence via the shared encoding “bandwidth” between VSTM and VLTM.

Relatedly, future studies should expand the generalizability of the “bandwidth” account. For instance, although our work examined a variety of VLTM that are consciously retrievable, not all VLTM are consciously retrievable (e.g., Chun & Jiang, 1998; Turk-Browne, Scholl, Chun, & Johnson, 2009). Is such implicit memory encoded with the same “bandwidth”? Although the finding by Turk-Browne and colleagues (Turk-Browne, Yi, & Chun, 2006) that attention serves as a common encoding factor for both explicit and implicit memory is suggestive of the common encoding “bandwidth,” future studies should directly assess this hypothesis. In addition, future studies should examine whether the “bandwidth” account generalizes to LTM encoding for other modalities. Given the limited capacity of STM in other modalities (Cowan, 2001; Gallace, Tan, Haggard, & Spence, 2008; Saults & Cowan, 2007; Visscher, Kaplan, Kahana, & Sekuler, 2007) and its relationship with attentional control (Ford, Pelham, & Ross, 1984; Van Hulle, Van Damme, Spence, Crombez, & Gallace, 2013; Wood & Cowan, 1995), our “bandwidth” account might provide a modality-general mechanism for LTM encoding.

## Footnotes

1 Although our experiments were inspired by the models of VSTM that incorporate discrete high-threshold representations, our predictions can be easily conceptualized in any VSTM models that acknowledge some form of capacity limit in the amount of visual information that can be represented precisely enough to inform mnemonic decisions.
